# A New Nanocomposite Copolymer Based On Functionalised Graphene Oxide for Development of Heart Valves

**DOI:** 10.1038/s41598-020-62122-8

**Published:** 2020-03-24

**Authors:** Evgeny A. Ovcharenko, Amelia Seifalian, Maria A. Rezvova, Kirill Yu. Klyshnikov, Tatiana V. Glushkova, Tatyana N. Akenteva, Larisa V. Antonova, Elena A. Velikanova, Vera S. Chernonosova, Georgy Yu. Shevelev, Darya K. Shishkova, Evgeniya O. Krivkina, Yuliya A. Kudryavceva, Alexander M. Seifalian, Leonid S. Barbarash

**Affiliations:** 1 Research Institute for Complex Issues of Cardiovascular Diseases, Kemerovo, Russian Federation; 2 UCL Medical School, University College London, London, United Kingdom; 3 Institute of Chemical Biology and Fundamental Medicine, Novosibirsk, Russian Federation; 4 NanoRegMed Ltd (Nanotechnology and Regenerative Medicine Commercialization Centre), London BioScience Innovation Centre, London, United Kingdom

**Keywords:** Cardiac device therapy, Valvular disease

## Abstract

Polymeric heart valves seem to be an attractive alternative to mechanical and biological prostheses as they are more durable, due to the superior properties of novel polymers, and have the biocompatibility and hemodynamics comparable to tissue substitutes. This study reports a comprehensive assessment of a nanocomposite based on the functionalised graphene oxide and poly(carbonate-urea)urethane with the trade name “Hastalex” in comparison with GORE-TEX, a commercial polymer routinely used for cardiovascular medical devices. Experimental data have proved that GORE-TEX has a 2.5-fold (longitudinal direction) and 3.5-fold (transverse direction) lower ultimate tensile strength in comparison with Hastalex (p < 0.05). The contact angles of Hastalex surfaces (85.2 ± 1.1°) significantly (p < 0.05) are lower than those of GORE-TEX (127.1 ± 6.8°). The highest number of viable cells Ea.hy 926 is on the Hastalex surface exceeding 7.5-fold when compared with the GORE-TEX surface (p < 0.001). The platelet deformation index for GORE-TEX is 2-fold higher than that of Hastalex polymer (p < 0.05). Calcium content is greater for GORE-TEX (8.4 mg/g) in comparison with Hastalex (0.55 mg/g). The results of this study have proven that Hastalex meets the main standards required for manufacturing artificial heart valves and has superior mechanical, hemocompatibility and calcific resistance properties in comparison with GORE-TEX.

## Introduction

Valvular heart disease (VHD) encompasses both acquired and congenital heart defects, which require proper surgical management. Surgical heart valve replacement, either with mechanical heart valve or tissue heart valve, remains to be one of the most commonly performed procedures. However, neither of these two types of commercially available artificial heart valves are ideal as they have their own limitations. Mechanical heart valves are commonly associated with a high risk of thrombosis and require patients to take anticoagulants, that can cause thromboembolic (1% per patient-year) and bleeding (0, 5% per patient-year) complications^1^. Tissue heart valves have limited durability, due to the degradation of the biological tissues used for their manufacturing^2,3^. The ideal replacement valve should offer physiological hemodynamic, life-long durability, without the need for anticoagulant therapy, and should not depend on the age-based cut off^4^. The concern of an age-based cut off as one of the key indications in selecting optimal heart valve prosthesis has risen dramatically in the last few decades. An increasing number of young adults are now being diagnosed with severe VHD in the developing world and the exponentially increasing ageing population requires life-saving procedures in developed countries^5^.

Polymeric heart valves (PHV) represent an attractive alternative to existing prostheses, since they can overcome major drawbacks of tissue and mechanical heart valves. Polymeric materials mimic the physiological manner of native heart valve leaflets whilst remaining simple for large-scale production. PHV is potentially suitable for rapidly evolving minimally invasive transcatheter devices and tissue engineering (TE) approaches^6^, the well-known key trends in today’s cardiac surgery. There are a number of different scaffolds that are made from biological and synthetic materials for TE applications^7^ and different fabrication techniques, including electrospinning^8^. However, their development and translation into clinical trials may take over 20 years. None of the current research in this field has yet neared the stages for satisfactory preclinical testing. Therefore, PHV might be the best option for immediate application^9^. Polymeric materials are relatively cheap, easily engineered and may withstand calcification and thrombosis, remaining biocompatible, strong and viscoelastic.

This has now necessitated a shift in the paradigm for heart valve design technology and associated materials towards elastomeric materials suitable for flexible heart valve leaflets. The drawbacks of the first-generation synthetic materials, biodegradation and thrombogenicity, have been overcome by advanced biocompatible elastomers^10^. The variation of the chemical structure of elastomeric polymers enables developing materials to mimic native tissue properties. Recent studies have reported their benefits over tissue heart valves, in terms of improved durability, and over mechanical heart valves, in terms of obviated need for life-long anticoagulation, thus ensuring excellent hemodynamic performance similar to that of native heart valves^4,11–14^.

Previously, Seifalian and co-workers developed a number of materials potentially suitable for cardiovascular devices and suppliers, including compliant polyurethane with a polycarbonate soft segment (PCU). This material was successfully commercialized and used for manufacturing vascular access grafts^15^. Studies showed that this vascular access graft possessed a compliance profile similar to human arteries and was more resistant to biodegradation than earlier versions of PU grafts^16^. The global vascular graft market size is valued at USD 209.1 million. It is likely to expand at a rate of 4.1% per year over the 2025 forecast period^17^. Despite the positive experience of using PCU for manufacturing vascular access grafts, it cannot be translated to the research and development of PHV. There are some significant differences in the fabrication process as well as in the mechanical properties. Heart valve prostheses should possess superior mechanical properties to resist shear stress, calcification and leaflet tearing. While vascular access grafts are usually used for a short-term period, until either a suitable donor is found or replaced, in the case of failure. Vascular grafts should have similar compliance to arteries in order to avoid distal intimal hyperplasia.

Taking into account these differences, Seifalian’s research group has developed a novel nanocomposite polyurethane with a polycarbonate soft segment (PCU) and polyhedral oligomeric silsesquioxane (POSS) specifically for the manufacturing of polymeric heart valves^18^. POSS–PCU demonstrated superior mechanical properties and was more hydrophobic compared to the original PCU material^19^. The POSS nanocomposite has had a number of successes and the polymer is currently under development for the application of a number of synthetic organs. However, recent studies demonstrated the lack of integration with the surrounding tissue, and a new material has been developed and patented based on fumed silica nanocomposite^20^.

**Figure 1:**
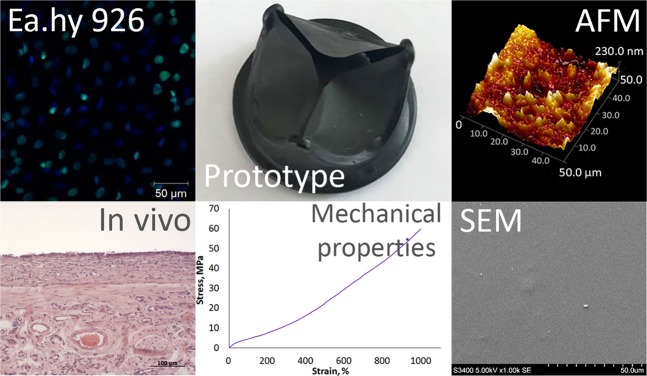
Summary of the main findings of the article.

Graphene has recently been of particular interest in the biomedical field due to its novel electrical, mechanical, optical, and thermal properties. Graphene monolayer is one atom thick, and the sp^2^ hybridization of C = C bonds are robust against the dislocation of carbon atoms under thermal variation, even at high temperatures. It is 200 times stronger than steel and, at the same time, viscoelastic^21^. We have developed a new nanocomposite material based on the integration of functionalised graphene oxide (FGO) nanomaterials into a backbone of poly(carbonate-urea)urethane (PCU) with the trade name Hastalex. Hastalex is already under development for tendons, urethra, facial organs and abdominal membranes. The material has a number of applications in other industries, including antifouling for marine application and for use as fibers in the textile industry. The aim of this study was to evaluate Hastalex for its possible application in the development of polymeric heart valves (Fig. 1). Therefore, physical and mechanical properties, biostability and hemocompatibility of Hastalex should be confirmed *in vitro* and *in vivo*.

## Material and Methods

### Materials

Two groups of materials were included in the study (1) Hastalex (NanoRegMed Ltd, London, UK)^22^ and (2) expanded polytetrafluoroethylene, GORE-TEX vascular graft (Gore & Associates, Inc., USA), a commercially available synthetic material commonly used for cardiac-related applications. GORE-TEX was used as a benchmark.

### Polymer synthesis Hastalex

The synthesis of Hastalex nanocomposite was previously described in detail^22^. In brief, graphene oxide was initially functionalised with the amine group. Then polycarbonate polyol and amine FGO were mixed and heated up to 70 °C to dissolve the FGO, forming the pre-polymer. The resultant material contained 5% of FGO. Dimethylacetamide was then added to the pre-polymer and was chain-extended by the addition of diethylamine. The polymer solution of 25% wt was produced. Films for further testing were obtained by polymer solution casting. As a result, samples had two unequal sides, one of them – shiny, which was in contact with the receiving surface, and the other one – opaque. All the chemicals were purchased from Aldrich Limited (Gillingham, United Kingdom).

### Mechanical properties

Uniaxial tensile testing was performed to estimate the mechanical properties of the Hastalex and GORE-TEX samples using the Static Materials Testing Machine Series Z with a 50 N load cell (ZwickRoell, Germany). All tests were performed in accordance with the editorial rules of the ISO 37:2017. The exact shape of the samples was cut using the cutting press ZCP 020 (ZwickRoell, Germany). The mechanical properties were evaluated quantitatively with the stress-strain analysis, including the estimation of the Young’s modulus (MPa), ultimate tensile strength (MPa), and uniform elongation (%) using TestXpert II Testing Software (ZwickRoell, Germany). Young’s modulus was measured within the physiological range of stresses. The testing was performed in the longitudinal (n = 5) and transverse (n = 5) directions for GORE-TEX due to orthotropic behavior and without choosing a direction for Hastalex (n = 8) due to isotropic behavior. To recalculate tension forces into normal stress, the samples thickness was measured using the mechanical thickness gauge with a clamping force of 1.5 N and a measurement error of ±0.01 mm.

### The contact angle measurements

The water adsorption on the surface of the polymer samples (Hastalex and GORE-TEX) was measured with the Drop Shape Analyzer DSA25 (Kruss GmbH, Germany). The contact angle was estimated by the sessile droplet method with five measurements on different areas of each polymer. All the tests were performed at a room temperature of 25 °C.

### Assessment of surface topography at micro- and nanoscale

#### Surface topography using atomic force microscopy (AFM)

The study and control samples of polymer samples (Hastalex, GORE-TEX) with area 0.5 × 0.5 cm^2^ were fixed with the specimen holders. The surface properties of the samples were estimated using the MultiMode 8 Atomic Force Microscope system (Bruker, Germany) in a tapping mode at ambient temperature and pressure. The SNL-10 probe (Bruker, Germany) was used in the study. Twenty images were obtained for each group of the samples. The data were processed with the Nanoscope Analysis 1.4 software (Bruker, Germany) to evaluate a 3D surface topography and height distribution profile.

#### Surface property using scanning electron microscopy (SEM)

Morphology and surface structure were evaluated using the scanning electron microscope S-3400N (Hitachi, Japan) under high vacuum mode at an accelerating voltage of 5 kV in the secondary electrons imaging mode. The samples were coated with a thin Au/Pd layer through sputter coating using vacuum system Emitech SC-7640 (Quorum Technologies, England).

### Assessment of biocompatibility using cell culture

The cytotoxicity tests were performed using established cell lines in accordance with ISO 10993-5: 2009 requirements. We chose endothelial cells Ea.hy 926. Ea.hy 926 is a hybridoma made by fusing human umbilical vein endothelial cells and a human A549 lung carcinoma cell line. These cells display the main phenotypic and functional characteristics related to the endothelium. The samples of Hastalex and GORE-TEX were sterilized with ethylene oxide. Cell culture plastic was used as a control since it supports the attachment, survival, and proliferation of adherent cells such as Ea.hy 926.

Cell viability and proliferation assays were carried out as in our previous study^23^. Briefly, Ea.hy 926 cells were cultured in a DME/F12 medium containing 1% HEPES buffer, 10% fetal bovine serum, 1% L-glutamine, 100 units/ml penicillin, 0.1 µg/ml streptomycin, 0.1 µg/ml amphotericin B, *hypoxanthine*-*aminopterin*-*thymidine* (Sigma Aldrich, USA) in a humidified atmosphere with 5% CO_2_ at 37 °C. The polymer matrices (n = 3/each group) were fixed at the bottom of a sterile 24-well culture plate with a 0.6% agarose solution (Helicon, USA). The cells were seeded on the samples at a density of 2.0 * 10^5^ cells/cm^2^ and cultured for 5 days. To assess cell viability, samples were stained with 2 μg/ml Hoechst 33342 (nuclei of adherent cells were shown in blue) (Sigma-Aldrich, USA) and 0.03 mg/ml ethidium bromide (nuclei of dead cells were shown in orange) (AppliChem, Spain). The polymer samples were separated from the agarose and transferred to the sterile 24-well plate with cells settling down. The total number of cells per 1 mm^2^ surface and the relative proportion of dead cells on samples were assessed using the fluorescence microscopy Axio Observer Z1 (Carl Zeiss, Germany).

Ea.hy 926 cell proliferation was assessed on the samples of matrices (n = 3/group) using the Click-iT Plus EdU Imaging Kits (Molecular probes, USA). The nuclei of the proliferating cells were stained with green-fluorescent Alexa Fluor 488 dye. When DNA is synthesized, thymidine is incorporated into DNA, and, having affinity with Alexa Fluor 488, allows for selective detecting the synthesized DNA with green-fluorescent nuclei. The nuclei of all cells were contrasted with DAPI. The number of stained cells was evaluated using the laser scanning microscope (LSM 700, Carl Zeiss, Germany).

### Assessment of hemocompatibility

Hemocompatibility tests were performed according to the requirements of ISO 10993-4: 2017.

Blood for the study was taken from healthy donors with written consent. To determine the level of erythrocyte hemolysis, fresh citrated blood was used in a 9:1 ratio (blood: sodium citrate). At the first stage of testing, GORE-TEX and Hastalex samples were incubated in 10 ml of physiological saline at a temperature of 37 °C for 2 hours. After that, 0.2 ml of citrated blood was added and the obtained mixture was incubated for 1 hour at a temperature of 37 °C, followed by centrifugation. The absorbance of supernatant was measured on a spectrophotometer GENESYS 6 (Thermo Fisher Scientific, USA) at a wavelength of 540 nm.

The hemolytic ratio (HR) was calculated according to formula^24^:

$${\rm{HR}}={\rm{Dt}}-{\rm{Dn}}/{\rm{Dp}}-{\rm{Dn}}\times 100 \% ,$$

where Dt are the absorption values for the experimental samples, and Dn and Dp are the absorption values for the negative and positive controls, respectively.

The complete absence of hemolysis was determined as the arithmetic mean of the optical density index when measuring saline solution with the citrate blood probe. 100% hemolysis was determined as the arithmetic mean of the optical density index for measuring citrated blood with distilled water.

To determine the maximum aggregation of blood platelets after contact with the test material, citrated blood was centrifuged at 1000 rpm for 15 minutes at room temperature to obtain platelet-rich plasma (PRP). To calibrate the instrument, platelet-poor plasma (PPP) was used, which was obtained by centrifuging citrate blood at 3500 rpm for 10 minutes at room temperature. The contact time of the test samples with platelet-rich plasma was 5 minutes. The measurements were carried out in a spontaneous mode without aggregation inducers with the restoration of Ca^2+^ ions on a platelet aggregometer APACT 4004 (LABiTec, Germany).

Study samples were incubated with 500 μl of platelet-rich plasma to determine platelet adhesion after contact. After that, the samples were washed and fixed in a 2% solution of glutaraldehyde. Then the samples were dehydrated in alcohol solutions with an increase in concentration from 30% to 100%. The surface of the materials was studied using scanning electron microscopy, the deformation index (DI) was calculated using the following formula^25^:

$$\begin{array}{ccc}{\rm{DI}} & = & ({\rm{quantity}}\,{\rm{of}}\,{\rm{type}}\,1\ast 1+{\rm{quantity}}\,{\rm{of}}\,{\rm{type}}\,2\ast 2+{\rm{quantity}}\,{\rm{of}}\,{\rm{type}}\,3\ast 3+{\rm{quantity}}\,{\rm{of}}\,{\rm{type}}\,4\,\times \,4\\  &  & +{\rm{quantity}}\,{\rm{of}}\,{\rm{type}}\,5\ast 5)/{\rm{Total}}\,{\rm{quantity}}\,{\rm{of}}\,{\rm{platelets}}.\end{array}$$

Table 1 shows the type of the platelet activation.

**Table 1:** List of the calcification of platelet activation.

Types	Characteristics
I	Round or discoid not deformed platelets
II	Dendritic platelets with early pseudopodia sticking out
III	Spread dendritic platelets with intermediate pseudopodia sticking out; congregating
IV	Flat platelets with cytoplasm expanding among pseudopodia
V	Cytoplasm fully spreads; the shape of Pseudopodia cannot be seen clearly

### *In vivo* biocompatibility assessment

Animal study was approved by the Local Ethical Committee of Research Institute for Complex Issues of Cardiovascular Diseases (ID 0432018 issued on 05/14/2018). All experiments were performed in accordance with the guidelines of the Local Ethical Committee of Research Institute for Complex Issues of Cardiovascular Diseases and regulations of EUROPEAN DIRECTIVE 2010/63/EU. Wistar young rats weighing between 55 and 70 g were employed in subcutaneous implantation trials. Three types of materials were investigated in the experiment: the study group Hastalex, the comparison group GORE-TEX, and the positive control group bovine pericardium tissue, fixed by 0.625% Glutaraldehyde solution (GA-pericardium). The sterile samples 0.5 cm × 0.5 cm in size were inserted into the subcutaneous pouches made on the dorsum of each of 15 animals. The samples remained implanted for 14 days (5 animals - foreign body reaction) or 60 days (5 animals - foreign body reaction, and 5 animals - calcification assessment). At 2 weeks and 2 months after implantation, rats were sacrificed under general anesthesia, any surrounding capsule was taken for histopathologic examination or calcification assessment.

#### Histopathology

The explants were fixed in 10% buffered formalin, pH 7.4, and processed for paraffin embedding for histopathologic examination using hematoxylin and eosin (H&E, Biovitrum, Russia) and Alizarin Red S (Reachem, Russia). The obtained histological preparations were viewed under a light microscope AXIO Imager A1 (Carl Zeiss, Germany). Tissue ingrowth was excluded as a criterion as these samples were nonporous, while capsule formation was used as a determinant of implant-host interaction. The foreign body response parameters were estimated according to international standards ISO 10993-6:2016 and comprised the presence and extent of a fibrous capsule; the number of foreign body giant cells (FBGCs); the number of neovascularization foci; fatty infiltration; and calcification; the presence and number of lymph nodes.

#### *In vivo* calcification

In order to determine the calcium content, the explanted specimens were dried and weighed. The dry samples were placed into 0.5 ml of 50% perchloric acid and kept at 150 °C until a clear solution was obtained. Hastalex and GA-pericardium samples were subjected to complete hydrolysis, GORE-TEX, remained stable. The quantity of total calcium concentration was quantified by inductively coupled plasma optical emission spectrometry with iCAP 6500 DUO spectrometer (Thermo Scientific, USA), and was expressed in milligrams per sample and extrapolated to µm/mg of dried tissue.

### Statistical analysis

The statistical analysis was conducted using STATISTICA 10.0 Software (StatSoft, Inc., USA). For the normal distribution verification we used the Kolmogorov–Smirnov test. Non-parametric Mann-Whitney U test and Student’s t-test (for normal distribution) were used to evaluate the statistical significance of differences. All the quantitative data are presented as medians, I and II quartiles, minimum and maximum for the groups with skewed distribution or as mean and standard deviation for the normal distribution. A p-value of < 0.05 was considered statistically significant.

## Results

### Mechanical properties

The median thickness of Hastalex samples was 0.61 (0.51; 0.53; 0.68; 0,72) mm. Thickness of GORE-TEX samples was uniform and accounted for 0.47 mm.

**Figure 2:**
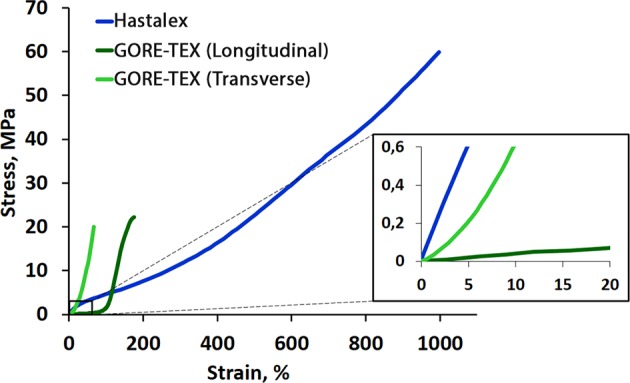
Stress-strain curves of Hastalex and GORE-TEX samples.

The orthotropic behavior of GORE-TEX was confirmed. Hastalex demonstrated equal mechanical properties in different directions, proving its isotropy (Table 2). GORE-TEX had a 2.5-fold (longitudinal direction) and 3.5-fold (transverse direction) lower ultimate tensile strength in comparison with Hastalex (p < 0.05) (Fig. 2).

**Table 2:** Mechanical properties of Hastalex (versus GORE-TEX).

	Hastalex (n = 8)	GORE-TEX (n = 5) Longitudinal direction	GORE-TEX (n = 5) Transverse direction
	Мe (25–75%; min-max)		
Ultimate tensile strength, MPa	57.1 (55.5–58.8; 51.4–59.3)	22.5 (20.4–24.1; 19.1–24.8)*	16.4 (15.8–16.8; 14.3–17.2)*
Uniform elongation,%	1004.3 (930.5–1059.8; 806.9–1089.6)	186.3 (183.0–191.1; 160.8–197.1)*	69.5 (63.5–77.9; 61.1–79.14)*
Young’s modulus, MPa	11.3 (10.4–12.3; 9.4–12.8)	1.9 (1.5–2.3; 1.1–2.45)*	10.1 (9.3–10.7; 9.1–10.9)

^*^р < 0.05 compared to Hastalex

The uniform elongation of Hastalex exceeded 5-fold greater than that of GORE-TEX in the longitudinal direction and almost 14-fold greater in the transverse direction (p < 0.05). However, Young’s modulus of Hastalex was similar to that of GORE-TEX in the transverse direction, but was 6-fold greater in the longitudinal direction (p < 0.05). The fiber structure of GORE-TEX lost its tensile strength with increased stiffness in the longitudinal direction, if large deformations (>100%) were applied. However, Hastalex continued to deform almost linearly.

### The contact angle measurements

**Figure 3:**
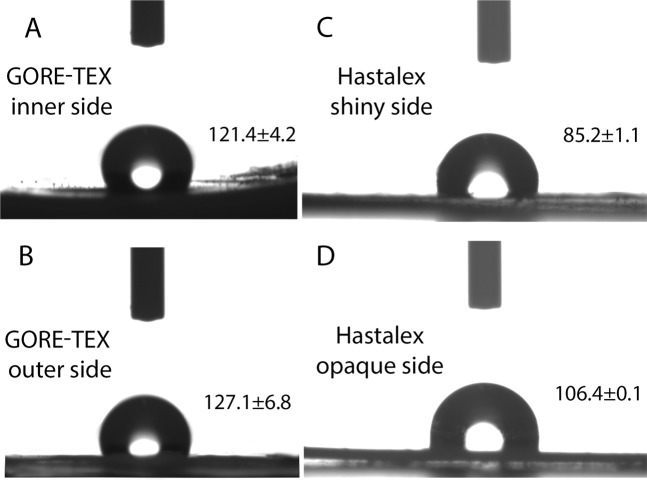
The water contact angle of Hastalex and GORE-TEX samples.

GORE-TEX had hydrophobic surfaces with average contact angles over 121.4 ± 4.2° (inner side) and 127.1 ± 6.8° (outer side), with no significant difference (p = 0.067) between both sides (Fig. 3).

Contact angle values of the opaque and shiny surfaces of Hastalex were significantly lower than those of GORE-TEX (p < 0.05). However, the shiny surface of Hastalex had a water contact angle of 106.4 ± 0.1° and was considered as hydrophobic, while the opaque surface had a water contact angle of 85.2 ± 1.1° and was referred to as hydrophilic. The difference between both sides had been detected (p < 0.05).

### Assessment of surface topography at micro- and nanoscale

#### Surface topography using atomic force microscopy (AFM)

**Figure 4:**
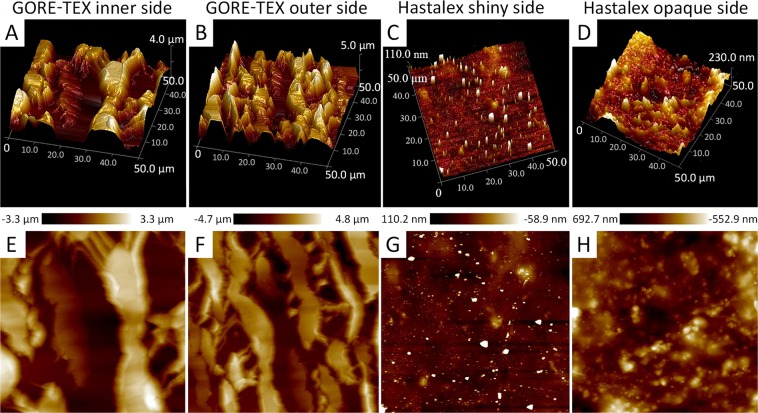
Typical AFM images of Hastalex and GORE-TEX on the top row and in the bottom 3D presentation of the data from the images.

The Hastalex AFM images (Fig. 4) demonstrated homogeneous surface structure for both sides of the polymer film (the height surface roughness did not exceed 0.3 μm but had irregular outliers of about 0.5 μm in height and about 10 μm in size). More irregular outliers with higher peaks on the opaque side were observed compared to the shiny side. However, these quantitative differences were insignificant. GORE-TEX exhibited regular and structured surface relief, except for 6 µm surface-height variations. The slopes varied from 20 to 30 μm. Differences in the topography of the inner and outer surfaces of the GORE-TEX had been determined by image analysis. Qualitative variations were observed for opaque and shiny sides of Hastalex. However, the quantitative description did not differ significantly for both sides.

#### Surface topography using scanning electron microscopy (SEM)

**Figure 5:**
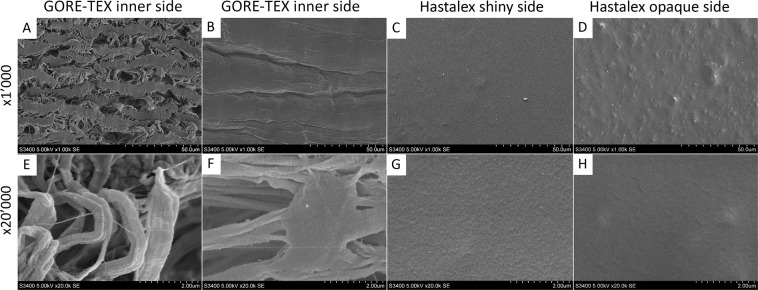
SEM images of GORE-TEX polymer matrices (**A,E**) – inner surface, (**B,F**) – outer surface; Hastalex (**C,G**) – shiny surface, (**D,H**)– opaque surface.

The SEM images (Fig. 5) showed that the GORE-TEX sample surface was heterogeneous and highly porous (Fig. 5E–H). In addition, the inner and outer GORE-TEX surfaces differed between each other, i.e. the inner surface (contacting with blood) had larger pores compared with its less porous outer surface. Similarly, the shiny surface of Hastalex (Fig. 5A,C) had fewer irregular defects (convexities) at ×1,000 magnification than the opaque one (Fig. 5B,D).

### Assessment of biocompatibility using cell culture

**Figure 6:**
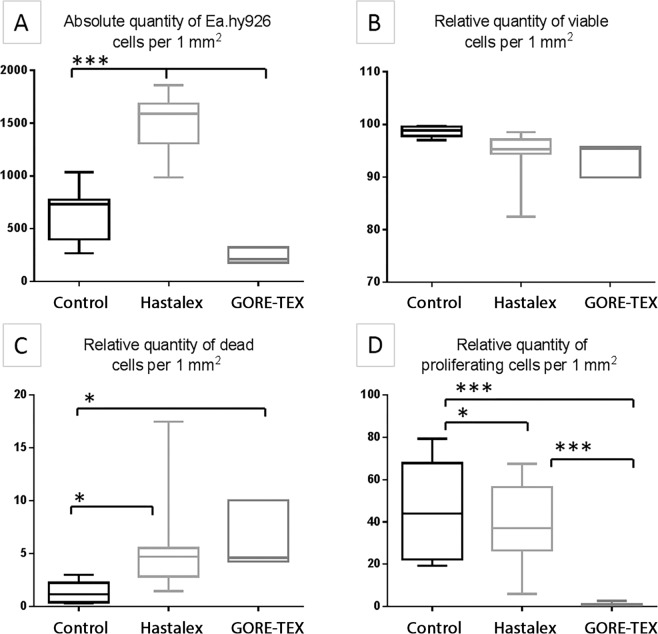
The number of Ea.hy 926 cells on Hastalex and GORE-TEX samples versus culture plastic after 5 days of cultivation: the absolute number of cells (**A**); relative number of viable cells (**B**); relative number of dead cells (**C**); the relative number of proliferating cells (**D**). *Significant differences between the groups, p < 0.05; ***significant differences between the groups, p < 0.001.

**Figure 7:**
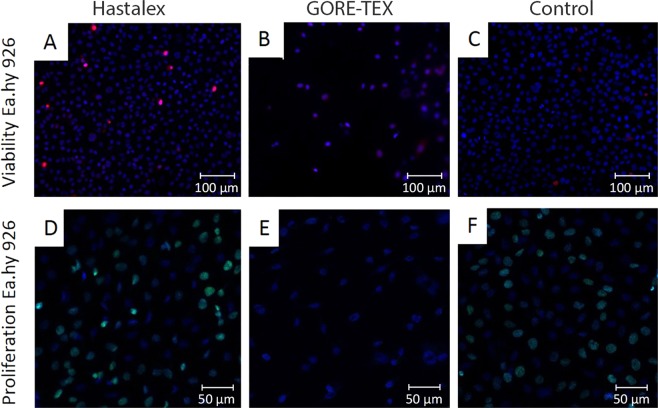
Fluorescence microscopy of Hastalex (**A,D**), GORE-TEX (**B,E**) and control (cell culture plastic) (**C,F**) with cells: staining for cell viability (**A–C**), ×200, staining for proliferation (**D–F**), ×200.

Five days after cultivation, the highest total number of viable cells Ea.hy 926 was determined on the Hastalex surface and accounted to 1,588 cells/mm^2^ (Fig. 6), exceeding 2.2-fold times the number on the control (p < 0.001) and 7.5-fold times the GORE-TEX surface (p < 0.001). There were no differences observed in the relative number of viable cells on the surface of the studied polymeric material and control (Figs. 6, 7) and varied in the ranges of 95.28% on the Hastalex surface to 98.84% on the cell culture plastic. The relative number of dead cells on the studied polymeric surfaces was 4.72% (Fig. 6), which was 4-fold higher than that on the cell cultural plastic (p < 0.05)

Cell proliferation/viability on the surface of biomaterials potentially allows for the indirect evaluation of biocompatibility of the material. In our experiments, the GORE-TEX surface demonstrated low cell adhesion due to the detachment of non-viable cells from the surface of the material. The *in vivo* staining of the remaining attached cells did not show any reliable difference in the viability relative to other polymer groups. Importantly, cell proliferation of the Ea.hy 926 line on the Hastalex surface was 37.10% similar to that of the indicator on the cell culture plastic (p < 0.05) and significantly exceeded proliferative activity of cells on the GORE-TEX surface (Figs. 6, 7).

### Assessment of hemocompatibility

According to the obtained data there was no negative effect on the RBC membranes for both types of test materials (Table 3). No statistically significant differences were noted in the degree of hemolysis between Hastalex and GORE-TEX samples (p = 0.60).

**Table 3:** Indicators of hemocompatible properties of Hastalex and GORE-TEX materials.

Type of material	The degree of hemolysis of red blood cells,%	Maximum Platelet Aggregation,%	Platelet Deformation index
	Мe (25–75%; min-max)		
Hastalex	0.4 (0.1–0.7; 0.1–0.7)	22.3 (18.9–22.7; 16.8–26.2)	1.9 (1.6–2.9; 0–3.7)
GORE-TEX	0.4 (0.3–0.7; 0.1–2.4)	26.8 (25.5–31.5; 22.9–38.9)	3.8* (3.3–4.0; 3.0–4.1)

^*^р < 0.05 compared to Hastalex.

The maximum platelet aggregation activity of intact blood platelet-rich plasma was 8.6% (25%: 8.0%; 75%: 10.1%; min: 7.8%; max: 15.9%). Analysis of the maximum platelet aggregation after contact with the test samples showed significantly higher values compared to intact PRP (Table 1) (p < 0.05), while there were no statistically significant differences between Hastalex and GORE-TEX (p = 0.62).

**Figure 8:**
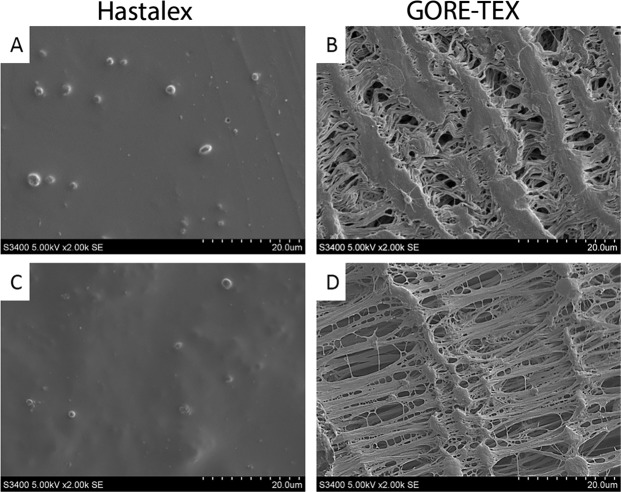
The platelet adhesion onto Hastalex surface (the shiny side - **A**, the opaque side - **C**) and GORE-TEX (inner side - **B**, outer side - **D**).

Assessment of blood platelet adhesion after contact with Hastalex and GORE-TEX showed the presence of adherent platelets on the surface of both polymer samples (Fig. 8), whilst the degree of platelet deformation on GORE-TEX was higher than that of the Hastalex surface. The platelet strain index of GORE-TEX was 2-fold higher than that of Hastalex (p = 0.03).

### *In vivo* biocompatibility assessment

#### Tissue response

**Figure 9:**
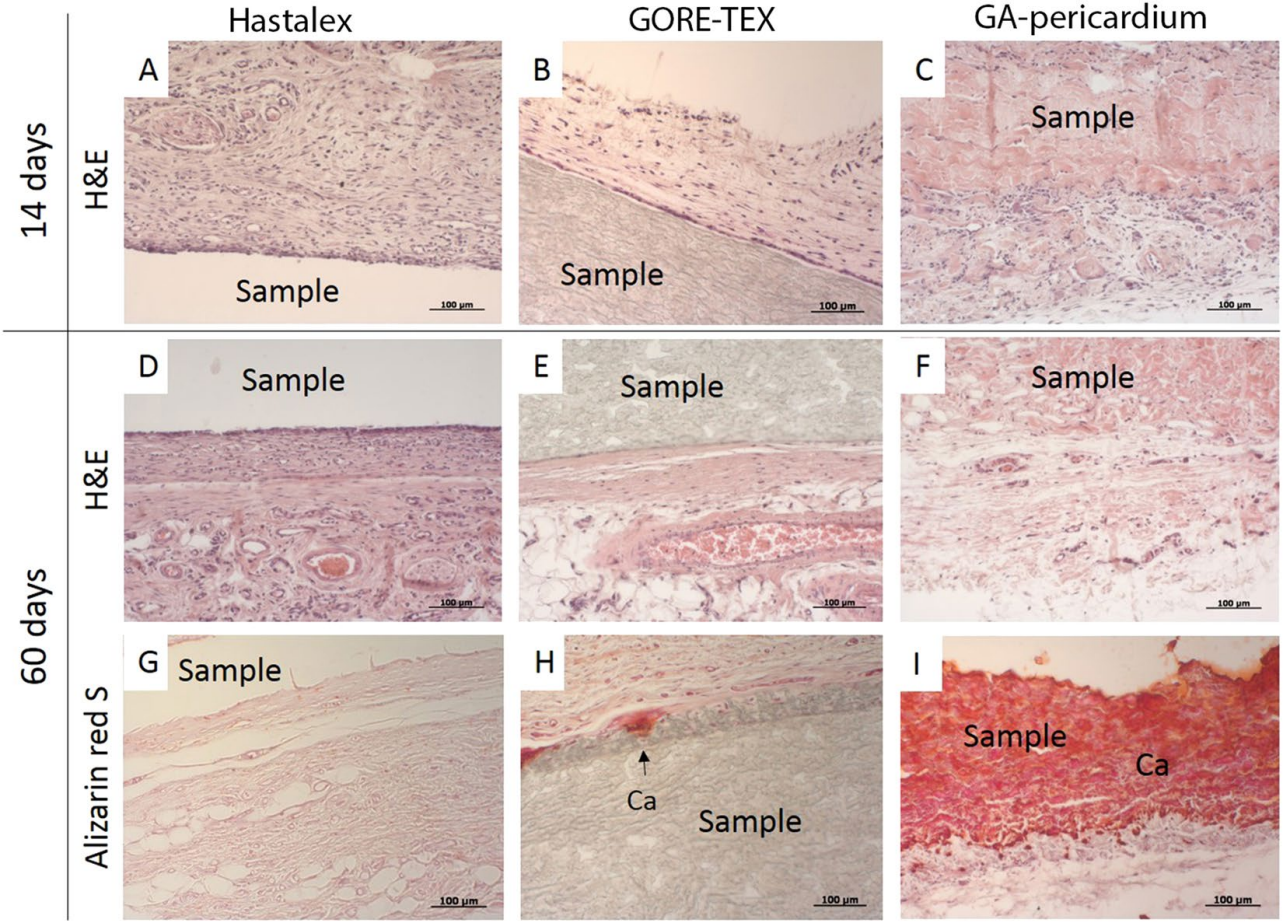
Histological sections (×200) of the Hastalex (**A,D,G**), GORE-TEX (**B,E,H**) and GA-pericardium (**C,F,I**) samples following a 14- (**A–C**) and 60-days (**D–I**) subcutaneous implantation in rats. Hematoxylin-eosin staining (**A–F**), alizarin red S staining (**G–I**).

Two weeks after implantation, a moderate macrophage infiltration had been detected. Microscopic examination of the tissues surrounding the implantation sites demonstrated no foreign-body giant cells and lymph nodes for all samples (Fig. 9A–C). The presence of the last, commonly suggests the onset of inflammation. The loose collagen-based fibrous capsule was observed around samples. The fibrous capsule thicknesses for the investigated materials were 133 µm (Hastalex), 69 µm (GORE-TEX), and 50 µm (GA-pericardium). Thus, fatty infiltration associated with collagen fibrils was observed in Hastalex and pericardium. Xenomaterial samples contained a higher number of fatty infiltrates. All samples were comparable in terms of neovascularization, i.e. minimal capillary proliferation, 1–3 neovessel formations with cells at ×400 magnification field. Cellular infiltration was noted in pericardium and GORE-TEX (due to the porous structure of the last).

Dense connective tissues with evident collagen fibers were observed in Hastalex (42 µm) and GORE-TEX (78 µm) 2 months after implantation (Fig. 9D,E). In the same period, active inflammation was noted for GA-pericardium samples due to the presence of a large number of giant cells and a wide network of neovascularization. A connective tissue capsule for GA-pericardium was not visualized. FBGCs were present in the fibrous capsules of the other two materials 2 months after implantation. The presence of these cells may be associated with the formation of calcium deposits (Fig. 9). The semi-quantitative comparison of the foreign body response performed according to the editorial rules and standards of the ISO 10993-6 allows the consideration of Hastalex as a biocompatible material.

#### *In vivo* calcification

Calcification resistance is one of the factors suggesting biocompatibility of artificial materials. It is a key factor determining the lifespan of heart valve prostheses. Staining with alizarin red S reported the presence of calcification, especially in the GA-pericardium group, with large amounts of calcium crystals visible within the explanted sample (Fig. 9I). Hastalex demonstrated more promising results. It had none or minimal calcium deposits of the connective tissue capsules (Fig. 9G). GORE-TEX samples contained visible calcific deposits at the tissue/implant interaction (Fig. 9H). GORE-TEX and GA-pericardium reported higher calcium concentration (p < 0.05) in comparison with Hastalex (Table 4).

**Table 4:** Calcium content in the explanted samples (mg/g) after a 60-day period: Hastalex, GORE-TEX and GA-pericardium.

	Min	25%	Me	75%	Max
Hastalex	0.49	0.50	**0.55**	1.08	1.57
GORE-TEX	6.2	7.0	**8.4**	9.1	9.2
GA-pericardium	131.1	134.8	**146.1**	182.1	216.3

A p-value of ≤ 0.05 was considered statistically significant. Reliable differences are determined for each group of the samples.

## Discussion

During the cardiac cycle, valve leaflets are exposed to three basic loading factors: shear stress, strain and fatigue. Flexure, occurring during valve opening, as opposed to tension, occurring when the valve is closed to prevent the reverse flow of blood, generally contributes to leaflet deformation^26^. Shear stress occurs when blood is passing through the open valve. Taking into account the fatigue of the material (prostheses perform almost 30 million cycles within one year)^27^, the mechanical properties of the leaflet material are of paramount importance. Energy loss should also be taken into consideration. In order to minimize energy loss and develop thin and durable leaflets, minimal energy for opening and closing, as well as certain coaptation.

Our results on the strength and elastic deformation properties of GORE-TEX are consistent with the literature data^28^. Hastalex is superior to GORE-TEX in terms of tensile strength that substantially exceeded the load, experienced by native valve tissues and vessels. However, not all artificial materials are capable of regeneration and will accumulate fatigue damage. This limitation means researchers must choose materials with improved tensile strength corresponding to the physiological loading required for fabricating PHV^29^. Special attention should be paid to the elastic deformation properties of Hastalex as a novel material for manufacturing PHV because despite its great ability to elongate under extreme loads, it appears to be a rather rigid material in the range of physiological load when compared with traditional biological materials used for the manufacturing of artificial heart valves^30^.

Hastalex has a much higher Young’s modulus than that of GORE-TEX in two mutually perpendicular directions, which is an important aspect in minimizing energy loss. The final hydrodynamic efficiency of Hastalex heart valve prostheses will largely depend on the design of its leaflets, but superior rigidity of this polymer with high deformability enables the manufacturing of thinner leaflets. Therefore, this material seems to be beneficial for manufacturing transcatheter heart valves. However, we should bear in mind that GORE-TEX samples have lower stiffness and anisotropy due to its fiber patterns as a result of the technology of its production. The original stiffness of the material is significantly higher, but its resultant porous structure is considered a negative factor in regards to biological inertness. Thus, non-porous films have certain advantages. Mechanical properties of original Hastalex enable its application in other medical devices: patches, artificial mitral chordae and vascular grafts. Nevertheless, its fatigue properties should be addressed in the further studies, since alterations in mechanical properties of the material may lead either to its tears and leaflet perforations (fatigue), or to its rigidity (calcification) and subsequent leaflet dysfunction.

Physical properties and body environment are well-known contributors to the thrombus formation caused by blood contact with synthetic (polymeric) materials. The ability of synthetic materials to adsorb proteins is the key factor promoting clotting during the first blood-material interactions. It is almost impossible to accurately predict and model protein adsorption due to the wide diversity in the blood system. However, we can assume with specific paths how the process may begin, with the knowledge of whether the surface is hydrophilic or hydrophobic^31^. Hydrophobic materials are generally considered to be more thrombogenic, since the adsorption of proteins is largely irreversible on their surface due to altered protein conformation. Hydrophilic materials are more hemocompatible due to the hydration layer formed on the surface and resulting in lower protein attachment^32^. The presence of aliphatic groups –CH_2_ – CH_3_, aromatic groups, and perfluoro groups (C_2_F_4_) determines the hydrophobicity of materials, whereas ionic groups (acidic or basic), polar chemical groups (ester R-COOR, ester –COC, alcoholic C-OH), zwitterionic polymers are hydrophilic^33^. Thus, there is some evidence on the successful application of the hydrophobic biomaterials (in particular GORE-TEX) for developing synthetic prosthetic heart valves^12^. However, with our previous experience, the optimal contact angle should be within the range of 85–100°^19^. Hastalex, a nanocomposite copolymer, is based on functionalised graphene oxide covalently bonded on the poly(carbonate-urea)urethane (PCU) polymer chain at prepolymer synthesis stage. Its hydrophobicity is imparted by the properties of functionalised graphene oxide, its concentration and the exact insertion of the polymer chain. For heart valve application we use contact angle order of 90°, which is less than that for GORE-TEX.

Amongst other factors, surface relief also determines hemocompatibility of the biomaterial. In addition to surface topography, biocompatibility of materials vary due to the electrostatic charge, chemical properties of the polymeric functional groups, molecular mobility, and amorphous/crystallinity, etc.^34,35^. There is some evidence suggesting that such chemical groups as –NH_2_, –OH, or –COOH can affect the activation of the complement system, leading to the covalent binding of C3b protein to one of the proteins of the complement cascade^36^. Even though the blood coagulation cascade and the complement system are usually considered independently from each other, these events are largely interdependent^37^.

**Figure 10:**
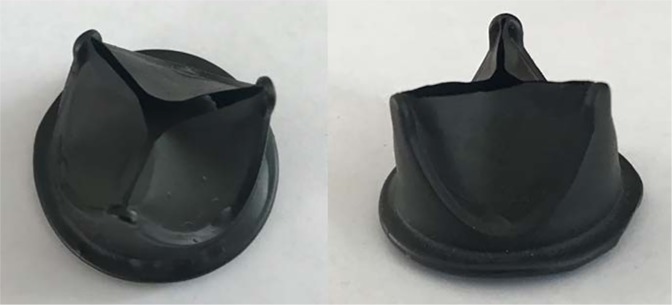
A prototype of polymeric heart valve made from Hastalex.

Chemical properties of biomaterials are of key importance for the manufacturing of vascular grafts with a small diameter and low blood flow, while topography and molecular mobility (surface layer chain mobility) are significant for blood flow in larger vessels^38,39^. The latter are also important in the manufacturing heart valve prostheses. Smoother and structured surfaces are known to cause a less pronounced inflammatory response than rough and porous surfaces. In addition, unstructured surfaces of synthetic materials can physically damage blood components, causing their adsorption and activation of protective blood coagulation systems^40^. Considering the biocompatibility of GORE-TEX in conjunction with a highly porous surface and roughness (Figs. 4, 5), we could conclude that Hastalex is potentially biocompatible due to the properties of its surface relief. However, the impact of polymer surface roughness on its biocompatibility should be considered separately for each polymer matrix, since biocompatibility can increase with reduced encapsulation rate in highly porous structures^28^. Irregular structural elements, pores and gas inclusions can accelerate biodegradation of the polymer and the product made from it^41^. This results in an increased risk of delayed dysfunction of the implants. The presence of convex elements (probably gas inclusions) on the opaque surface of Hastalex, confirmed by the SEM findings (Fig. 5), requires further studies as well as the selection of optimal conditions for manufacturing polymer matrices, which can be used for manufacturing prosthetic heart valves (Fig. 10).

Platelet adhesion occurs as a result of different surface charges and is the initial stage of the thrombus formation. However, platelet adhesion cannot solely trigger thrombogenic reactions, i.e. only activated platelets are able to release into the blood substances that catalyze irreversible aggregation. Type I platelets were commonly found on the Hastalex surface (Fig. 8). Type I platelet adsorption is reversible and less likely to cause coagulation and thrombosis, since such platelets can easily be returned to the bloodstream due to their regular round shape, without any deformations^24^. According to RBC hemolysis, Hastalex and GORE-TEX can be considered to be hemocompatible, since the extension of RBC hemolysis following contact with the polymer surface did not exceed 0.4%, with acceptable hemolysis rates of up to 2%^42^. Our data is consistent with the results of other recent studies^43^.

Our experiment demonstrated low cell adhesion onto the GORE-TEX surface, which is generally consistent with previous studies^44^. The cytotoxicity test displayed that Hastalex had high biocompatibility, showing high cell adhesion, viability and proliferation. Cell adhesion and proliferation play an essential role for tissue-engineered heart valves. Taking into account the obtained results, we may conclude that Hastalex has certain potential in the development of tissue-engineered implants.

The implantation of any foreign material inevitably provokes a host response. The formation of connective tissue capsule indicates the end of the inflammatory process. The thickness of the fibrous capsule characterizes the degree of the material biocompatibility. At 60 days, the fibrous capsule around Hastalex was thinner than that around GORE-TEX, suggesting advanced biocompatibility of the former. FBGC, found on the explanted samples, are capable of secreting reactive oxygen species and other chemical agents, potentially contributing to oxidative damage and destruction of the implanted devices^45^.

Early calcification is one of the key mechanisms resulting in severe stenosis of native and tissue heart valves prosthesis. Similarly, it is a major limitation in the development of PHV^46^. Polymers, apart from xenomaterials, lack mineralization provoking phosphorus-rich cellular debris and destroyed collagen, emphasizing their relatively superior resistance to this process. Calcium concentration in the GA-treated xenopericardial sample, chosen as a positive control, was consistent with other studies^47^, and was significantly higher than those values for two study polymers. There are two major types of mechanisms that provoke calcification following the implantation of synthetic material: (1) deposition of calcium-phosphate crystals onto the implant surface or at the implant/capsule interface; and (2) calcification of biological tissue formed around the implant. The first mechanism (deposition of calcium crystals onto the sample surface) was observed in the GORE-TEX sample, assigned to the comparison group, whereas the Hastalex sample showed calcific deposits formed at the fibrous capsule. According to the quantitative analysis, Hastalex has superior resistance to calcification when compared to GORE-TEX. This finding suggests that Hastalex may be used for the manufacturing PHV. In addition, there is a large body of evidence proving the tendency of GORE-TEX towards calcification in clinical experiments^11^.

## Conclusion

The data showed that Hastalex, a graphene-based nanocomposite material, is the new contender for the development of heart valve leaflets. The results of this study have proven Hastalex to meet the criteria required for manufacturing artificial heart valves. It has superior mechanical properties, hemocompatibility and calcific resistance. Hastalex has greater mechanical strength, reduced platelet adhesion and lower calcium absorption in comparison with GORE-TEX. Both polymers are comparable in terms of biocompatibility. GORE-TEX has shown optimal safety with a high level of evidence in numerous studies and is now widely used for manufacturing cardiovascular medical devices. GORE-TEX is currently the only commercially available material applied in heart valve prostheses. Obtained data have confirmed that Hastalex properties are superior to those of GORE-TEX. Our study provides the basis for further *in vivo* experiments to estimate long-term biocompatibility and biostability of Hastalex as a heart valve prosthesis. The heart valve, both open-heart surgery and transcatheter, is a multi-billion dollar industry with huge interest to clinician, academia and industries.
